# Heat and Mass Transfer with Condensation in Capillary Porous Bodies

**DOI:** 10.1155/2014/194617

**Published:** 2014-01-28

**Authors:** Salah Larbi

**Affiliations:** Laboratory of Mechanical Engineering and Development, Department of Mechanical Engineering, Polytechnic National School of Algiers, 10, Avenue Hassen Badi, B.P. 182, El-Harrach, 16200 Algiers, Algeria

## Abstract

The purpose of this present work is related to wetting process analysis caused by condensation phenomena in capillary porous material by using a numerical simulation. Special emphasis is given to the study of the mechanism involved and the evaluation of classical theoretical models used as a predictive tool. A further discussion will be given for the distribution of the liquid phase for both its pendular and its funicular state and its consequence on diffusion coefficients of the mathematical model used. Beyond the complexity of the interaction effects between vaporisation-condensation processes on the gas-liquid interfaces, the comparison between experimental and numerical simulations permits to identify the specific contribution and the relative part of mass and energy transport parameters. This analysis allows us to understand the contribution of each part of the mathematical model used and to simplify the study.

## 1. Introduction

Transport phenomena in porous media with phase change take an important part in simultaneous heat and mass transfer process. They are encountered in many applications, in industrial problems as well as in natural situations [1]. A typical case of these applications is the vapour condensation in construction walls in which moisture absorption has very harmful consequences on thermal and mechanical properties in the material used.

It is well known when a porous structure is in contact with hot and humid air in one side and with an impermeable and cold wall in another side and under specific conditions in temperature and humidity a condensation process can appear in this structure and consequently thermal insulation properties of the material used as well as mechanical properties of the structure can be affected.

Although the mathematical modelling of heat and mass transport in porous media was studied several years ago [1–6], the mathematical approaches and assumptions used remain without any validation in many cases. Most published theoretical works are based on assumptions related to liquid phase which is in pendular or in funicular state cases [6–13].

Experimental studies have been performed for consolidate materials cases containing different pores size [14] or where the liquid phase is considered as continuous, such as in drying processes [15–18]. In our knowledge, few experimental studies have been conducted for dry porous medium cases [19–21]. The importance of these analyses are related to understanding the liquid phase distribution, the mathematical modelling of these phenomena, and the legitimacy in using the classical continuous description models by considering the diffusion coefficients as based on phases continuity determination. Assumptions related to liquid phase continuity are generally justified in drying processes but are not necessarily acceptable below a certain saturation degree, particularly in initially dry medium.

It should be noted that the diffusion coefficients characterizing the porous structure and used in the mathematical models are given, starting from specific experiments relating to an initial saturation of the condensate, and when the liquid phase is considered as continuous. This situation which is commonly encountered when the liquid phase is set up by damping is not necessarily representative of situations in which condensation is carried out in initially dry medium [22].

Udell [23] investigated the effects on a porous layer, which contains water, of heating the layer at the top and cooling the layer from below and at one-dimensional study case. Experimental results show that, at steady state, there are three distinct zones within the porous pack: a vapour zone at the top, a liquid zone at the bottom, and a two-phase zone in between. In the two-phase zone there is a counter flow of liquid, driven upwards by capillary forces, and vapour, driven downwards by a pressure gradient. The one-dimensional steady-state heat and mass transfer in a two-phase zone of a water-saturated porous medium is studied. The system consists of a sand-water-vapour mixture in a tube that is heated from above and cooled from below.

Bridge et al. [24] presented an extension to existing models of the two-phase zone by adding an energy equation to the system considered by Udell and assuming an explicit temperature dependence for the vapour pressure. Their analysis is extended to allow for variations in temperature throughout the two-phase zone of a three-zone system.

Ogniewicz and Tien [25] rigorously studied condensation phenomenon in porous media where the coupling between temperature and concentration of condensing vapour was taken into account. Motakef and El-Masri [26] were interested in the one-dimensional transport analysis of heat and mass with phase change in a porous slab, and analytical solutions for the cases of immobile and mobile condensate were obtained.

Shapiro and Motakef [27] proposed an analytical solution for a large class of transient problems and compared their results with experimental data. Wyrwa and Marynowicz [28] have used the approach of Motakef and El-Masri for the problem of one-dimensional flow of heat and diffusion of vapour in a porous wall. Heat and vapour transfer with condensation in a porous wall is analytically investigated. Notice that very little work exists on the particular subject of vapour condensation in porous materials.

Glaser's [29] primarily thermal diffusion model is based on Fick's law and it is still used widely in civil engineering, for condensation risk analysis and in defining the quality specifications which the constructive elements must satisfy, due to its simplicity of use graphically. It only lets the unidirectional moisture transfer intervene in the vapour phase and assumes a steady state. This method allows one to predict condensation. However, it considers that the liquid phase resulting from condensation does not have any subsequent movement. The comparison of the theoretical and experimental results [30] shows that Glaser's theory is insufficient to predict the condensation phenomena.

The Krischer-Vos [31] model represents the first attempt to describe the moisture transfer in porous materials while keeping in mind both the vapour and liquid phases. However, the transport in the liquid phase depends exclusively on the humidity gradient and neglects the temperature gradient. This method is no better than Glaser's for the calculation of condensed quantities, even though it allows calculation of the size of the condensed zone.

Many authors have presented numerical solutions of heat and mass transfer equations corresponding to the mathematical model proposed by Crausse [32]. Most of these studies are in relation to partially defined environments or elements made up of only one layer, splitting up elements with different characteristics. The solution of equations applied to multiply layered walls has been less used, due to the greater difficulty in translating the conditions of continuity existing in the interfaces of the different layers.

The different mechanisms of moisture transport in building walls and the analysis of interface phenomena have been investigated by de Freitas et al. [33]. Their mathematical model is based on the mathematical model proposed by Luikov and Philip-De Vries, where a computer program has been developed. The comparison of calculated and measured values obtained by using gamma-ray equipment to measure water content is in a good agreement with those obtained theoretically.

Vapour diffusion through the building elements and the wetting by condensation have been subject to theoretical and experimental research having as a basis Glaser's method and Vos's method. Larbi [30] and Crausse [32], among others, have shown by comparing the results obtained by experimentation with the results obtained by solving the heat and moisture transfer equations that the Luikov and Philip- De Vries models have fewer drawbacks for the prediction of condensation and the distribution of humidity in the interior of porous materials than the models of Glaser and Vos.

The porpose of the work presented in this paper is related to an analysis of condensation phenomena taking place in a capillary porous body and in a half open system, by solving governing equations derived from the mathematical model used, by giving the distributions of heat and moisture content in this media and by validating these results by experimental data.

## 2. Mathematical Modelling

### 2.1. Governing Equations

The fluid flow model used is based on conservative balance equations (mass, momentum, and energy). The governing equations describing simultaneous heat and mass transfer in porous media that we aim to test in our study related to condensation phenomena case are given by [6]

(1) $$\begin{array}{c} \frac{\partial \omega }{\partial t}=\nabla \cdot \left(D_{\omega }\nabla \omega +D_{T}\nabla T+D_{G}\nabla z\right), \end{array}$$

for mass conservation equation and

(2) $$\begin{array}{c} {\left(\rho C\right)}^{*}\frac{\partial T}{\partial t}=\nabla \cdot \left(\lambda^{*}\nabla T\right)+\rho_{o}\Delta h_{v}\left[\nabla .\left(D_{\omega_{v}}\nabla \omega +D_{T_{v}}\nabla T\right)\right] \end{array}$$

for energy conservation equation.

With

(3) $$\begin{array}{c} {\vec{J}}_{m}=D_{\omega }\nabla \omega +D_{T}\nabla T,\\ {\vec{J}}_{v}=D_{\omega_{v}}\nabla \omega +D_{T_{v}}\nabla T,\\ {\vec{J}}_{l}=D_{\omega_{l}}\nabla \omega +D_{T_{l}}\nabla T,\\ {\vec{J}}_{m}={\vec{J}}_{v}+{\vec{J}}_{l}, \end{array}$$

where *J*_*m*_, *J*_*v*_, and *J*_*l*_ are, respectively, the total mass flow, the vapour, and the liquid mass flow. Structural properties as well as diffusion coefficients in the above model are determined experimentally [32].

### 2.2. Initial and Boundary Conditions

**Figure 1:**
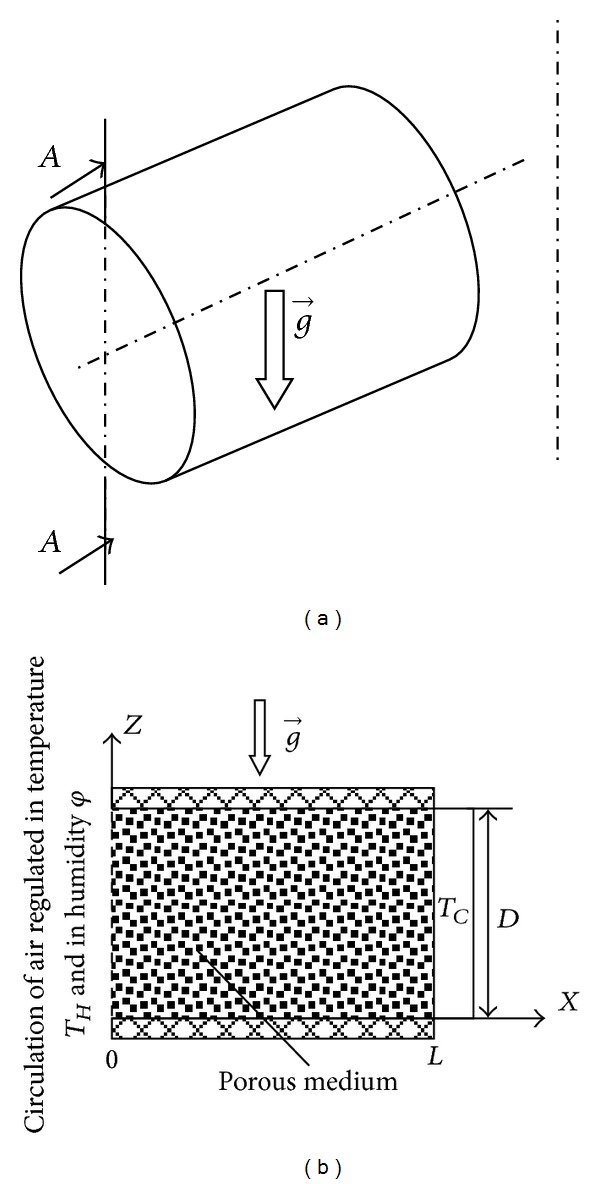
Physical model and coordinates system. (a) Physical model; (b) Section *A*-*A* showing the detail of the model used.

Schematic representation of the problem is given in Figure 1. To yield this study as simple as possible either in experimental or in numerical point of view, physical system that we aim to study is half open, with its open part (*x* = 0) in contact with an air regulated in temperature and in humidity the close one in (*x* = *L*) is maintained at constant temperature (*T*_*C*_) and lateral parts are insulated and closed.

#### 2.2.1. Initial Condition

Initially, the sample of the porous medium is a dry state, with uniform temperature (*T*_0_) and moisture content (*ω*_0_):

(4) At  t≤0 ω=ω0=constant, T=T0=constant.  

#### 2.2.2. Boundary Conditions

From the cold side, the wall is impermeable (mass flow null) and maintained at constant temperature. From the warm side, the evolution with time of the mass (*M*) of condensate water is determined by experimental measurements.

The conditions on the border, *x*, are given by

(5) At  x=0  and  for  (0<z<D),Jm=1ρohm(PvH−Pvw)=1ρoϕ˙m(t), T=TH,At  x=L  and  for  (0<z<D) Jm=0, T=TC,with  ϕ˙m(t)=ddt(ϕm(t))=1S·ddt(M(t))=4πD2·ddt(M(t)).

The conditions on the border, *z*, are given by

(6) At  z=0, z=D  and  for  (0<x<L) Jm=0, q=0.

## 3. Numerical Procedure

The mathematical model used in describing simultaneous heat and moisture transfer in porous medium, given by (1) and (2), is composed of two nonlinear partial differential equations of parabolic type. This system is solved numerically by using the finite element method with an integral formulation of GALERKIN type [34]. A computer program using FORTRAN language is then developed. Initial and boundary conditions are given by (4)–(6). The domain of resolution has a rectangular form. The chosen element is a quadrilateral element with four nodes.

## 4. Results and Discussion

The results are related to temperature and moisture content distributions in a porous medium. The sample of porous medium considered in the study is made of sand of almost uniform grain size diameter (100 < *d* < 125 *μ*m). The choice of this material is done to take into account our knowledge related to its structural properties and diffusion coefficients determined experimentally [32]. These elements will help us to test the validity of theoretical models named previously.

Experimental conditions used are hot air temperature, *T*_*H*_ = 30°C; water cold temperature, *T*_*C*_ = 10°C; air humidity, *φ* = 75% (regulation of humidity is done with the sodium chloride); initial temperature of the porous medium, *T*_0_ = 30°C; initial moisture content of the porous medium, *ω*_0_ = 0.02% (dry medium).

**Figure 2:**
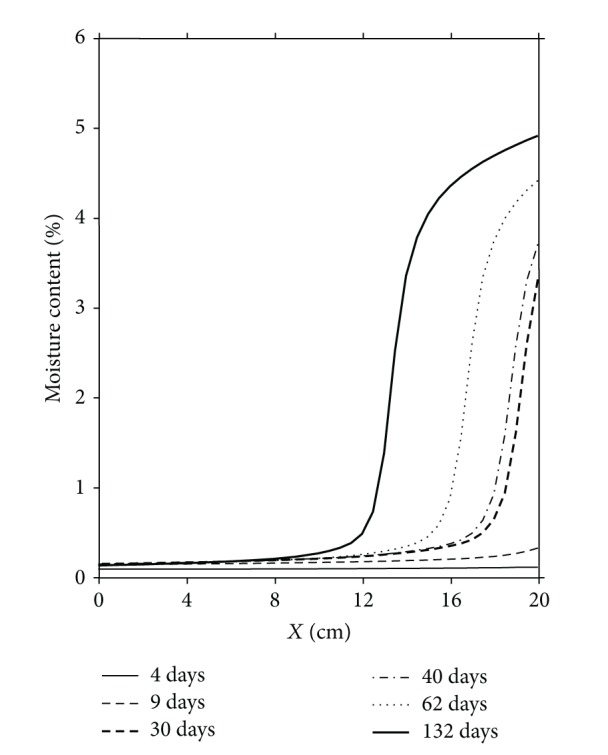
Distributions of moisture content. Numerical simulation results.

Figure 2 shows the distribution of moisture content obtained by numerical simulation at different times. It will be noted that the dry zone of the porous medium is in hygroscopic equilibrium with its environment and no point of condensation is observed in this zone although the dew point is located in it. At the first time of this study we have expected that the condensation phenomena can be located at a section corresponding to the dew point in the medium; the experimental results confirm the opposite. This result can be explained by evaporation-condensation process taking place simultaneously in this section. The wet zone appears on the cold side of the medium (*x* = 20 cm) and the front of condensation extends from this position towards (*x* = 12 cm).

**Figure 3:**
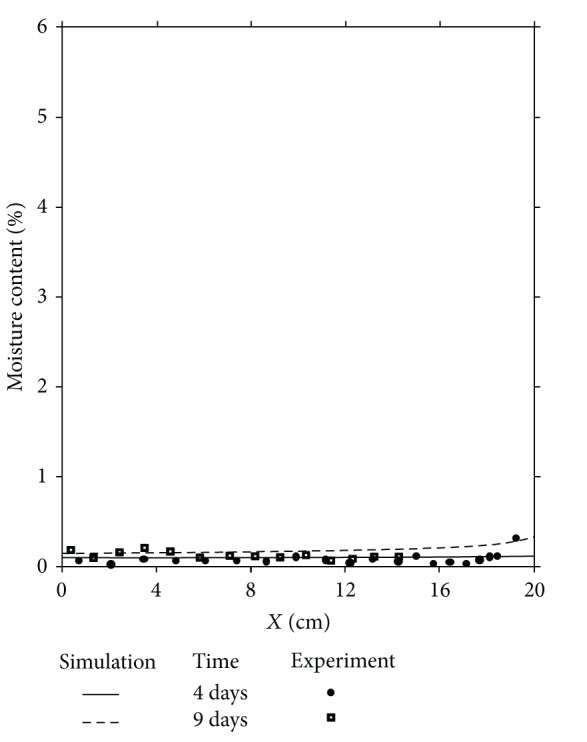
Moisture content distributions for *t* = 4 days and *t* = 9 days. Comparison between experiment and numerical simulation.

**Figure 4:**
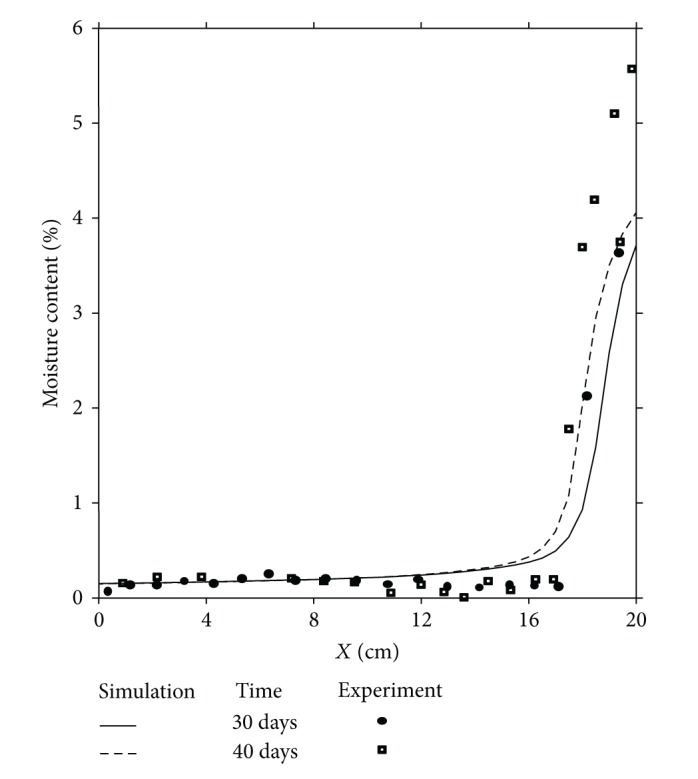
Moisture content distributions for *t* = 30 days and *t* = 40 days. Comparison between experiment and numerical simulation.

**Figure 5:**
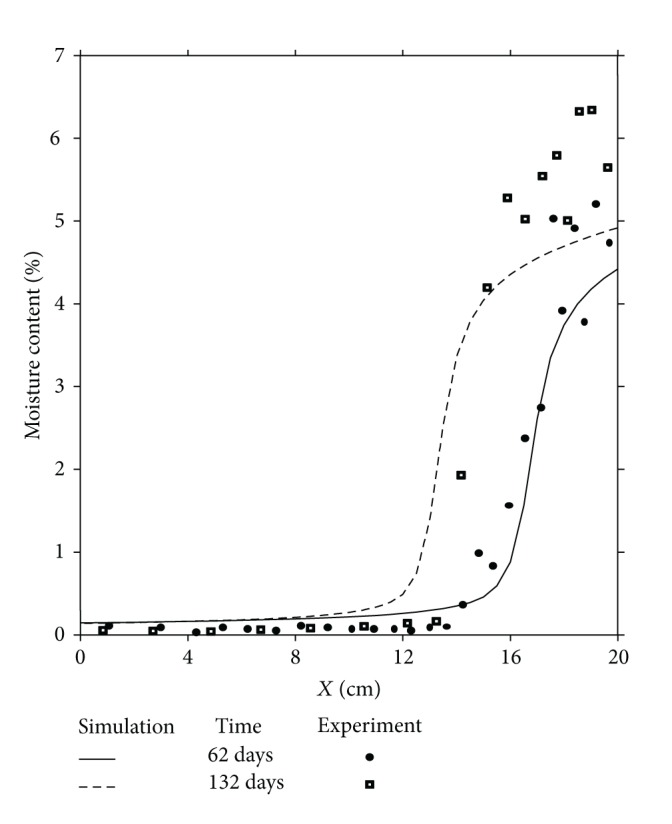
Moisture content distributions for *t* = 62 days and *t* = 135 days. Comparison between experiment and numerical simulation.

In order to validate the numerical simulation results a comparison is done between these results and those obtained experimentally [32]. Figures 3, 4, and 5 illustrate the comparison between experimental and numerical results related to the distributions of moisture content in one dimension of space, respectively, for 4 days and 9 days, 30 days and 40 days and finally 62 days and 135 days.

This comparison shows a good agreement between all these results; the approach of the mathematical model agrees well but in its qualitative form. However, if the qualitative aspect shows a good agreement between the physical reality and numerical simulation, the quantitative aspect presents a difference between these results, specially near the cold side (*x* = 20 cm) where the liquid phase due to the condensation phenomenon appears and where the moisture content is at its maximum value. This difference is due to the gravity effect, not taken into account in this comparison, as it is observed and explained by other authors [26]. To confirm this conclusion, a two-dimensional study of the problem is required by taking into account the term corresponding to the gravity effect in the mathematical model.

**Figure 6:**
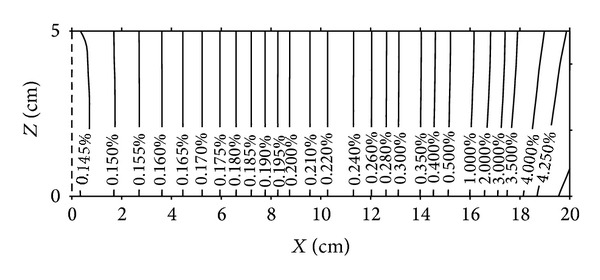
Moisture content distributions after 62 days.

**Figure 7:**
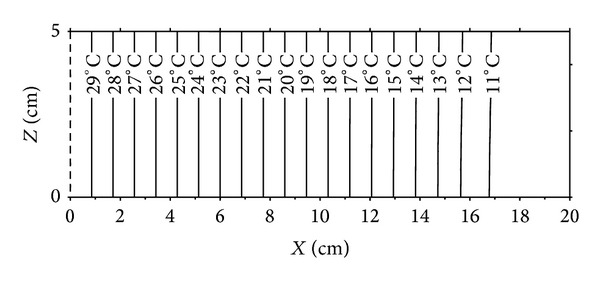
Temperature distributions after 62 days.

**Figure 8:**
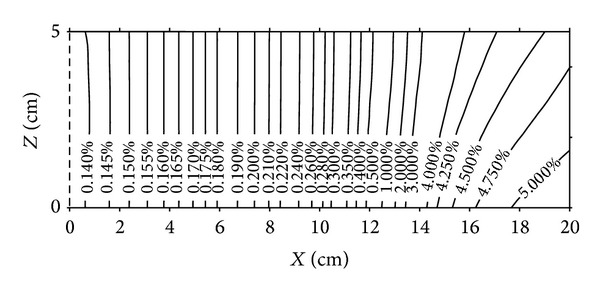
Moisture content distributions after 135 days.

**Figure 9:**
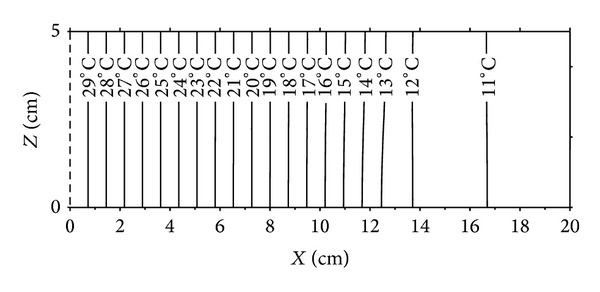
Temperature distributions after 135 days.

Figures 6, 7, 8, and 9 show the distributions of temperature and moisture content in two dimensions of space and at times 62 days and 135 days. It can be noted, at a first time, that for the temperature case, isothermal lines are vertical in the medium and for the moisture content profiles, the lines corresponding to the equal moisture remain vertical only for low values of them. Near the cold wet side, we observe light deformations of lines having values higher than 3% of moisture content. Thus the influence of the two dimensional aspect of moisture content distributions due to the gravity effect in the wet zone is then proved.

**Figure 10:**
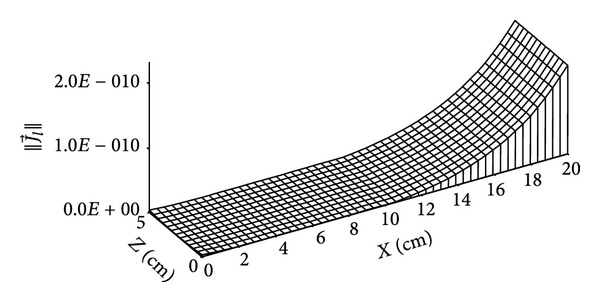
Distribution of liquid mass flow (in kg/m^2^·s) for *t* = 4 days.

**Figure 11:**
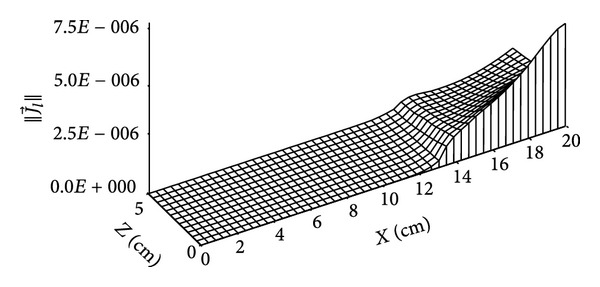
Distribution of liquid mass flow (in kg/m^2^·s) for *t* = 135 days.

**Figure 12:**
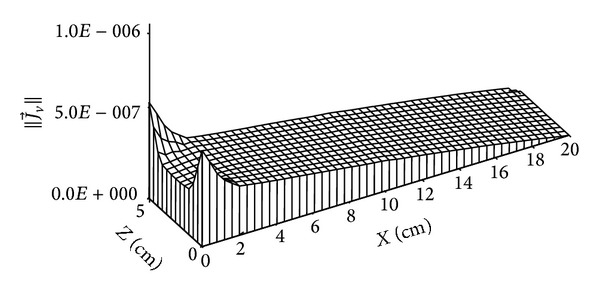
Distribution of vapour mass flow (in kg/m^2^·s) for *t* = 4 days.

**Figure 13:**
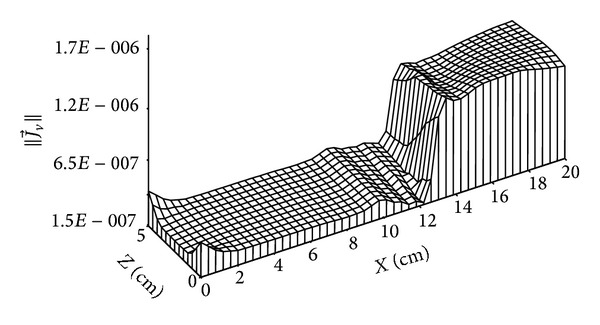
Distribution of vapour mass flow (in kg/m^2^·s) for *t* = 135 days.

**Figure 14:**
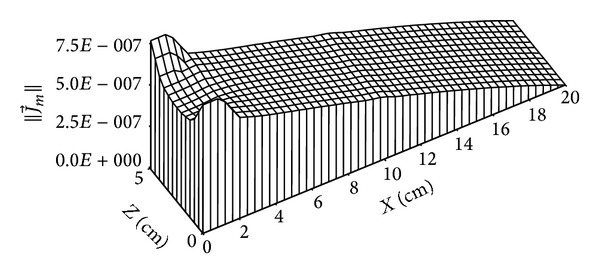
Distribution of total mass flow (in kg/m^2^·s) for *t* = 4 days.

**Figure 15:**
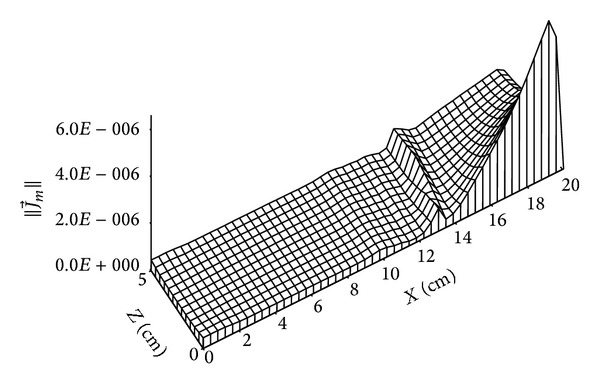
Distribution of total mass flow (in kg/m^2^·s) for *t* = 135 days.

Figures 11 to 15 give the distributions of mass flow at 4 days and 135 days. Figures 10 and 11 show the distribution of liquid mass flow where the displacement of this flow from the wet zone (*x* = 20 cm) towards the dry one (*x* = 0 cm) is due to capillary effects.

The distribution of vapour mass flow is given by Figures 12 and 13; this flux due to the vapour pressure gradient between external flow of humid air and dry medium moves from (*x* = 0 cm) to (*x* = 20 cm) until this vapour pressure gradient will be null and then no phenomenon of liquid mass flow occur.

The distribution of total mass flow is given by Figures 14 and 15. This total mass flow has two components: the liquid mass flow that is negative and the vapour mass flow that is positive. It will be noted that at low times, this flow is dominated by the vapour flow component.

**Figure 16:**
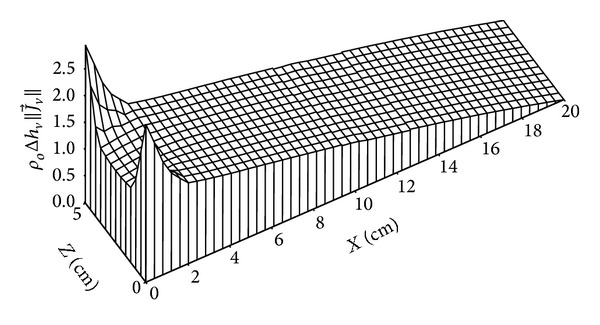
Distribution of heat flow due to phase change (in W/m^2^) for *t* = 4 days.

**Figure 17:**
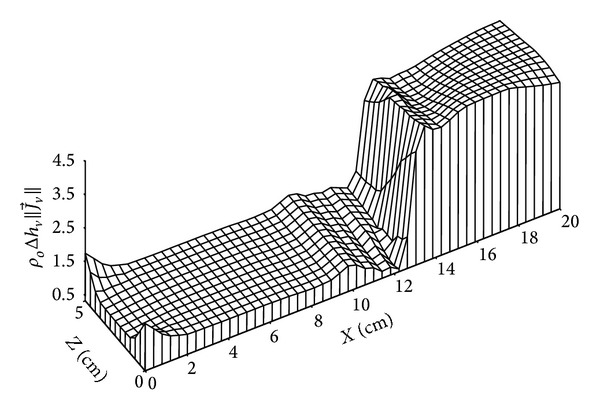
Distribution of heat flow due to phase change (in W/m^2^) for *t* = 135 days.

**Figure 18:**
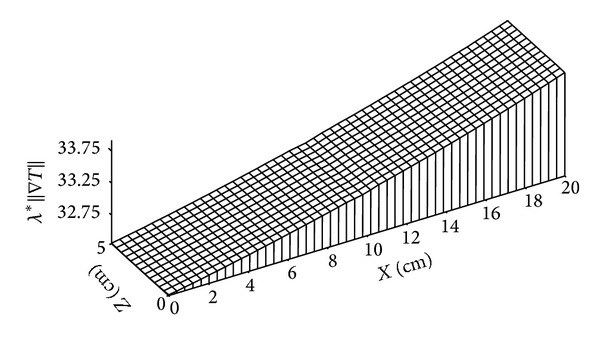
Distribution of heat flow due to conduction (in W/m^2^) for *t* = 4 days.

**Figure 19:**
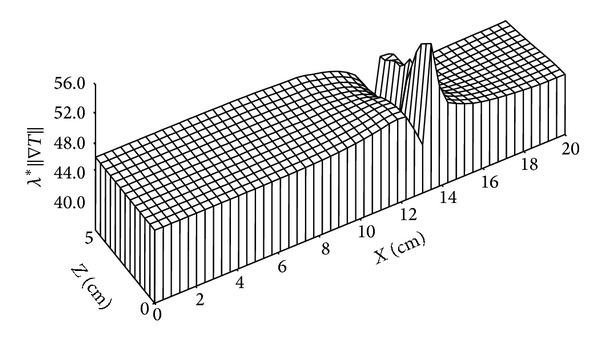
Distribution of heat flow due to conduction (in W/m^2^) for *t* = 135 days.

Figures 16, 17, 18, and 19 give the distributions of heat flow. It will be noted that the quantitative comparison between the heat flow due to the phase change and the heat flux due to conduction heat transfer shows a prevalence of the last one compared to that due to phase change. The mathematical model used can then be simplified, where the term corresponding to heat transfer due to phase change can be neglected in comparison with conductive heat transfer term.

## 5. Conclusions

The aim of the present work is related to heat and mass transfer analysis with condensation in capillary porous bodies initially dry. The considered physical system is half open, with its open part in contact with an air regulated in temperature and in humidity; the close part is maintained at constant temperature which is less than the saturation temperature and lateral parts are insulated and closed.

The presented results are related to temperature and moisture content distributions obtained experimentally and numerically. These results show that

1. the dry zone is in hygroscopic equilibrium with its environment and no point of condensation is observed in it;
2. the wet zone appears on the cold side of the medium and the front of condensation extends from this position towards the open side by capillary effects;
3. the comparison between experimental and numerical results shows a well description of the mathematical model used but in its qualitative form;
4. the amount of heat flow due to phase change is less important than heat flux due to conduction heat transfer;
5. the mathematical model on a macroscopic scale gives qualitative satisfying results in a half open system, due to the existence of an impermeable wall. Further studies can be extended to macroscopic as well as to microscopic scales and in open system cases without impermeable wall in order to understand the physical nature of the condensation process in structures.

## Nomenclature

*C*: Specific heat, J/kg·K
*D*_*T*_: Nonisothermal mass diffusion coefficient, m^2^/s·K
*D*_*G*_: Coefficient characterising gravity, m/s
*D*_*ω*_: Isothermal mass diffusion coefficient, m^2^/s
*D*: Diameter of the cell, m
*T*: Temperature, K
*L*: Lengh of the cell, m
Δ*h*_*v*_: Enthalpy of phase change, J/kg
*t*: Time, s
*x*, *z*: Coordinates of space
*h*_*m*_: Mass transfer coefficient, 1/s
*J*: Mass flow density, kg/m·s
*K*: Permeability, m^2^
*U*: Filtration velocity, m/s
*P*: Pressure, Pa
*Q*: Heat flow density, W/m^2^
*M*: Mass flow, kg/s
*S*: Surface of the cell, m^2^.

### Greek Symbols

*ω*: Moisture content, kg/kg
*λ*: Thermal conductivity, W/m·K
*ρ*: Density, kg/m^3^
*ε*: Porosity, m^3^/m^3^
*μ*: Dynamic viscosity, kg/m·s
*φ*: Relative humidity, %.

### Subscripts

*H*: Hot
∗: Porous medium
0: Initial
*a*: Dry air
*c*: Cold
*g*: Gas
*l*: Liquid
*m*: Mass
*o*: Apparent
*w*: Wall
*v*: Vapour.
